# Functional Characterization of Replication-Associated Proteins Encoded by Alphasatellites Identified in Yunnan Province, China

**DOI:** 10.3390/v14020222

**Published:** 2022-01-24

**Authors:** Liling Zhao, Xuan Che, Zhanqi Wang, Xueping Zhou, Yan Xie

**Affiliations:** 1 State Key Laboratory of Rice Biology, Institute of Biotechnology, Zhejiang University, Hangzhou 310058, China; zhaolilingyunnan@163.com (L.Z.); 21816001@zju.edu.cn (X.C.); zzhou@zju.edu.cn (X.Z.); 2 Biotechnology and Germplasm Resources Institute, Yunnan Academy of Agricultural Sciences, Kunming 650223, China; 3 Key Laboratory of Vector Biology and Pathogen Control of Zhejiang Province, College of Life Sciences, Huzhou University, Huzhou 313000, China; zhqwang@zju.edu.cn; 4 Key Laboratory for Biology of Plant Diseases and Insect Pests, Institute of Plant Protection, Chinese Academy of Agricultural Sciences, Beijing 100193, China

**Keywords:** alphasatellite, replication-associated protein, geminivirus complex, PTGS, TGS

## Abstract

Alphasatellites, which encode only a replication-associated protein (alpha-Rep), are frequently found to be non-essential satellite components associated with begomovirus/betasatellite complexes, and their presence can modulate disease symptoms and/or viral DNA accumulation during infection. Our previous study has shown that there are three types of alphasatellites associated with begomovirus/betasatellite complexes in Yunnan province in China and they encode three corresponding types of alpha-Rep proteins. However, the biological functions of alpha-Reps remain poorly understood. In this study, we investigated the biological functions of alpha-Reps in post-transcriptional gene silencing (PTGS) and transcriptional gene silencing (TGS) using 16c and 16-TGS transgenic *Nicotiana benthamiana* plants. Results showed that all the three types of alpha-Rep proteins were capable of suppressing the PTGS and reversing the TGS. Among them, the alpha-Rep of Y10DNA1 has the strongest PTGS and TGS suppressor activities. We also found that the alpha-Rep proteins were able to increase the accumulation of their helper virus during coinfection. These results suggest that the alpha-Reps may have a role in overcoming host defense, which provides a possible explanation for the selective advantage provided by the association of alphasatellites with begomovirus/betasatellite complexes.

## 1. Introduction

RNA silencing in plants is a natural defense system to counter against virus infections [1], which can occur at both transcriptional and post-transcriptional levels. Transcriptional gene silencing (TGS) in plants involves de novo RNA-directed DNA methylation, which is a well-characterized epigenetic mark conserved in plants, animals, and some fungi [2,3]. Post-transcriptional gene silencing (PTGS), which is initiated by double stranded RNA (dsRNA), is common in plant-virus interactions and is an evolutionarily conserved mechanism that protects host cells against invasive nucleic acids, such as viruses, transposons, and transgenes [4,5,6]. As a counter to these host defenses, most plant viruses encode proteins that act as suppressors of TGS and PTGS [7], such as Rice stripe virus (RSV) [8], Tobacco etch virus (TEV) [9], Cucumber mosaic virus (CMV) [10] and Cotton leaf curl Multan virus (CLCuMuV) [11].

The geminiviruses constitute a large family of single-stranded DNA pathogens that infect a wide variety of plant species and cause significant crop losses [12,13,14]. Many monopartite begomoviruses are now known to be associated with betasatellite or/and alphasatellite molecules [15,16,17,18,19]. Betasatellites, which are also known as DNAβs, have been well-studied in recent years. Betasatellites are circular single-stranded DNAs (ssDNAs) that are about half the size (~1.35 kb) of their helper virus genomes and can induce typical symptoms during infection [20,21,22]. Betasatellites are completely dependent on helper begomoviruses for their replication, systemic infection and transmitted by whiteflies [14,22]. They encode a single βC1 protein in the complementary strand, which acts as a symptom determinant and a viral suppressor of RNA silencing [23,24,25]. Alphasatellites are another type of satellite associated with begomoviruses, which have previously been known as DNA1s, and share no sequence homology with their helper viruses and the betasatellites. Like betasatellites, alphasatellites are also circular ssDNA molecules and are frequently associated with Old World begomovirus/betasatellite complexes [18,26,27]. They have a highly conserved genome organization, consisting of a single open reading frame (ORF) coding for a replication protein (Rep) with similarity to those of nanoviruses (another family of circular ssDNA viruses): an adenine-rich region of nearly 200 nucleotides and a predicted hairpin structure with the sequence TAGTATTAC which is considered to be the origin of replication (Ori) of nanoviruses [26]. Recently, alphasatellites have been found to be associated with New World bipartite begomoviruses. They are related to nanoviral DNA components and show a typical genome organization with an ORF coding, potentially for a Rep-associated protein [28]. Alphasatellites can autonomously replicate their own genomes but depend on their helper begomovirus for systemic infection, encapsidation, and vector transmission [29]. Some alphasatellites have been reported to modulate viral symptoms and/or affect the accumulation of their helper virus and/or betasatellite DNA during infection [30,31,32,33]. However, the precise biological functions of alphasatellites remain unclear.

It has shown that China has hosted more than 25% of geminivirus species and 50% of alphasatellite and betasatellite species [34]. Previously, we have reported that alphasatellites in China are classified as three types: Y10 alphasatellite, Y35 alphasatellite, and Y132 alphasatellite, according to their phylogenetic relationships [18]. Y10 alphasatellite, also named Y10DNA1, was associated with Tomato yellow leaf curl China virus (TYLCCNV)/Tomato yellow leaf curl China betasatellite (TYLCCNB) complex, Y35 alphasatellite was associated with Tobacco curly shoot virus (TbCSV)/Tobacco curly shoot betasatellite (TbCSB) complex, and Y132 alphasatellite was associated with TbCSV/Ageratum yellow vein betasatellite (AYVB) complex. In the present study, we isolated and investigated the biological functions of these three types of replication proteins encoded by alphasatellites (alpha-Reps) identified in Yunnan province, China. Our results showed that alpha-Reps are involved in the PTGS and TGS in transgenic *N. benthamiana* 16c and 16-TGS plants. We also found that alpha-Reps could enhance the accumulation of their helper virus, whereas the intact alphasatellite molecules have the opposite effects. Our findings give insights into the biological functions of alpha-Reps and may provide a new idea for the study of alphasatellites and their disease complexes.

## 2. Materials and Methods

### 2.1. Plant Materials and Growth Conditions

Wild-type *N. benthamiana* plants, *N. benthamiana* line 16C containing an active 35S-green fluorescent protein (GFP) transgene [35], and *N. benthamiana* line 16-TGS, which contains a transcriptionally silenced GFP transgene [36], were grown in an insect-free chamber or in limited access growth rooms at 25 °C with a 16:8-h (light/dark) photoperiod, as described in previous research [37].

### 2.2. Viral Genomic DNA Extraction and Amplification of Alpha-Rep Genes

Genomic DNA isolated from virus-infected *N. benthamiana* leaves was carried out as described in previous research [25]. Amplification of *alpha-Rep* genes from Y10, Y35 and Y132 isolates using PCR was performed as described in previous research [18]. Primers used for gene amplification are listed in Supplementary Table S1.

### 2.3. Sequence Analysis of Alpha-Reps

The amino acid sequence alignment was performed on alpha-Reps using the Clustal Omega software (https://www.ebi.ac.uk/Tools/msa/clustalo/ (accessed on 16 November 2019)) and visualized using the GenDoc software (http://gendiapo.sourceforge.net/ (accessed on 16 November 2019)), as described in previous research [38]. Conserved domains were predicated using the Pfam (http://pfam.xfam.org (accessed on 16 November 2019)) and Simple Modular Architecture Research Tool (http://smart.embl-heidelberg.de/ (accessed on 16 November 2019)), as described in previous research [39,40].

### 2.4. Recombinant pCHF3 Vectors for PTGS Suppression Analysis

To transiently express *alpha-Rep* genes, the ORFs were cloned into the *Bam*HI-*Cla*I sites of the binary vector pCHF3 [41]. All the recombinant pCHF3 vectors were introduced into the *Agrobacterium tumefaciens* strain C58C1 by the electroporation method. *A. tumefaciens* harboring recombinant pCHF3::TYLCCNV-βC1 (Tomato yellow leaf curl China virus βC1) or TBSV-P19 (Tomato bushy stunt virus P19) constructs were used as positive controls. *A. tumefaciens* harboring a binary expression plasmid carrying a full length GFP insert (35S::GFP) was kindly provided by Dr. D.C. Baulcombe, John Innes Center, UK).

### 2.5. Recombinant PVX Vectors for TGS Suppression Analysis

To generate the PVX expression constructs, the ORFs of *alpha-Rep* genes were cloned into the *Cla*I-*Sal*I sites of the PVX vector pGR106 [42]. All the recombinant PVX vectors were introduced into the *A. tumefaciens* strain GV3101 by the electroporation method. *A. tumefaciens* harboring an empty PVX vector (PVX::Vec) or a recombinant PVX::TYLCCNV-βC1 were used as negative and positive controls, respectively.

### 2.6. Viral Inoculation and Agroinfiltration

For PTGS and TGS experiments, *A. tumefaciens* cultures carrying designed constructs were infiltrated into 16c or 16-TGS *N. benthamiana* leaves as described in previous research [37,41]. For recombinant PVX vectors expressing *alpha-Rep* genes, each *A. tumefaciens* culture was adjusted to an optical density at 600 nm (OD_600_) of 0.8 before infiltration into 16-TGS *N. benthamiana* plants, as described in previous research [25].

For co-inoculation with TYLCCNV, the infectious clone of TYLCCNV was equally mixed with an *A. tumefaciens* culture carrying pCHF3::Y10alpha-Rep, pCHF3::Y35alpha-Rep, or pCHF3::Y132alpha-Rep, respectively, and then infiltrated into wild-type *N. benthamiana* plants as described in previous research [25,37]. Infectious clones of TYLCCNV alone, or co-inoculated with the infectious clone of Y10DNA1, TYLCCNB were used as the controls. Each *A. tumefaciens* culture was adjusted at an OD600 of 1.0 as described in previous research [25], and each treatment was infiltrated into 20 *N. benthamiana* seedlings. At 30 days post-inoculation (dpi), the uppermost systemic leaves per plant were collected and three plants were combined into one DNA sample for Southern blot analysis. These experiments were performed on three independent biological replicates.

### 2.7. RNA Extraction and Northern Blot Analysis

RNA isolation from infiltrated areas of leaves was performed using a TRIzol reagent (Invitrogen, Carlsbad, CA, USA). Total RNA (10–15 μg) was separated on 1.2% formaldehyde-agarose gels before being transferred to Hybond-N^+^ membranes (GE Healthcare, Bucks, UK) using 20× SSC buffer. The membranes were hybridized and detected as described in previous research [43].

### 2.8. Protein Extraction and Western Blot Analysis

Total proteins were extracted from leaf samples (approximately 100 mg) using 200 μL extraction buffer containing 50 mM Tris–HCl (pH 6.8), 9 M urea, 4.5% SDS, and 7.5% β-mercaptoethanol, as described in previous research [41]. The homogenates were shaken repeatedly on ice for 30 min and centrifuged at 13,000 rpm for 15 min at 4 °C, and the resulting supernatants were used for Western blot analysis. Proteins were transferred to nitrocellulose membranes after being separated by electrophoresis in 12.5% SDS-PAGE, and GFP was detected using an α-GFP (Epitomics, Burlingame, CA, USA) antibody, as described in previous research [25].

### 2.9. Southern Blot Analysis

For southern blot analysis, viral genomic DNAs were separated on a 0.8% agarose gel in Tris-borate-EDTA (TBE) buffer, and then transferred to Hybond-N^+^ membranes (Amersham Biosciences, Piscataway, NJ, USA). After that, the viral genomic DNAs were hybridized with digoxin-labeled probes using a DIG High Prime DNA Labeling and Detection Starter Kit II (Roche Diagnostics, Mannheim, Germany), as described in previous research [33]. Primers used for Southern blot analysis are listed in Supplementary Table S1.

## 3. Results

### 3.1. Isolation and Sequence Analysis of Three Types of Alpha-Reps from Yunnan Province, China

**Figure 1:**
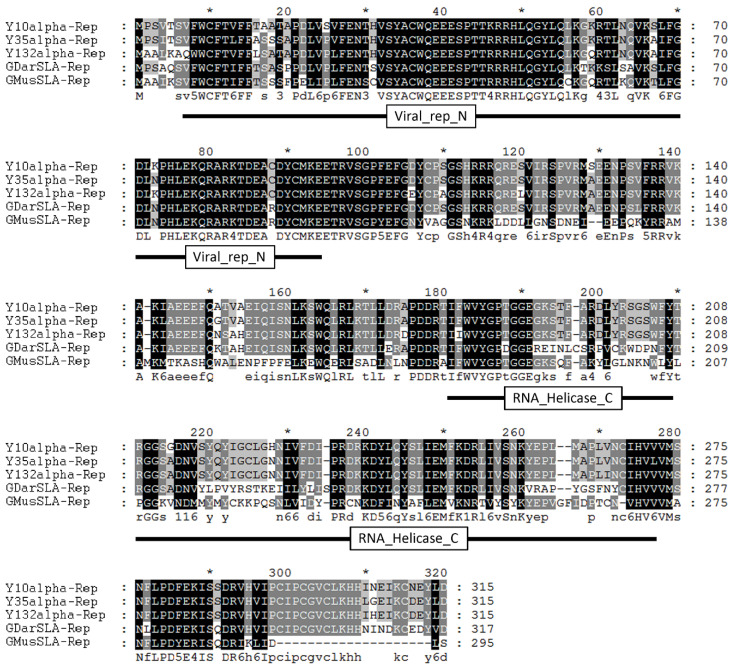
Sequence analysis of alpha-Reps. Amino acid sequence alignment of alpha-Reps of Y10 alphasatellite (AJ579353), Y35 alphasatellite (AJ579345), Y132 alphasatellite (AJ579349), GDarSLA (*Gossypium darwinii* symptomless alphasatellite, NC_013013), and GMusSLA (*Gossypium mustelinium* symptomless alphasatellite, NC_013012). Positions with strictly conserved amino acids are highlighted in black, conserved substitutions in dark gray, and blocks of similar residues in light gray.

Our previous study showed that there are three types of alphasatellites: Y10 alphasatellite, Y35 alphasatellite, and Y132 alphasatellite, associated with monopartite TYLCCNV/TYLCCNB, TbCSV/TbCSB, and TbCSV/AYVB complexes, respectively, in Yunnan province in China, which encode three corresponding types of alpha-Rep proteins (Y10alpha-Rep, Y35alpha-Rep, Y132alpha-Rep) [18]. According to the sequence information from our previous study, we designed gene-specific primers and obtained the whole ORFs of three types of *alpha-Rep* genes from Yunnan province, China (GenBank accession nos. AJ579353, AJ579345, and AJ579349). The coding region of these three types of *alpha-Rep* genes is 948 bp in length and encodes proteins of 315 amino acids (Figure 1). To examine the evolutionary relationship among alpha-Reps from various alphasatellites, we performed an amino acid sequence alignment. The result showed that Y10alpha-Rep, Y35alpha-Rep, Y132alpha-Rep, GDarSLA-Rep (Rep encoded by *Gossypium darwinii* symptomless alphasatellite, GDarSLA), and GMusSLA-Rep (Rep encoded by *Gossypium mustelinium* symptomless alphasatellite, GMusSLA) display a high sequence similarity (48.0–93.0%) (Figure 1 and Table S2). Conserved domain analysis based on the Pfam (http://pfam.xfam.org (accessed on 16 November 2019)) and Simple Modular Architecture Research Tool (http://smart.embl-heidelberg.de/ (accessed on 16 November 2019)) indicated that alpha-Rep proteins have a Viral_rep domain and an RNA_helicase domain in their N and C termini, respectively (Figure 1), suggesting that they are replication-related proteins.

### 3.2. Alpha-Reps Are Suppressors of Local GFP PTGS

**Figure 2:**
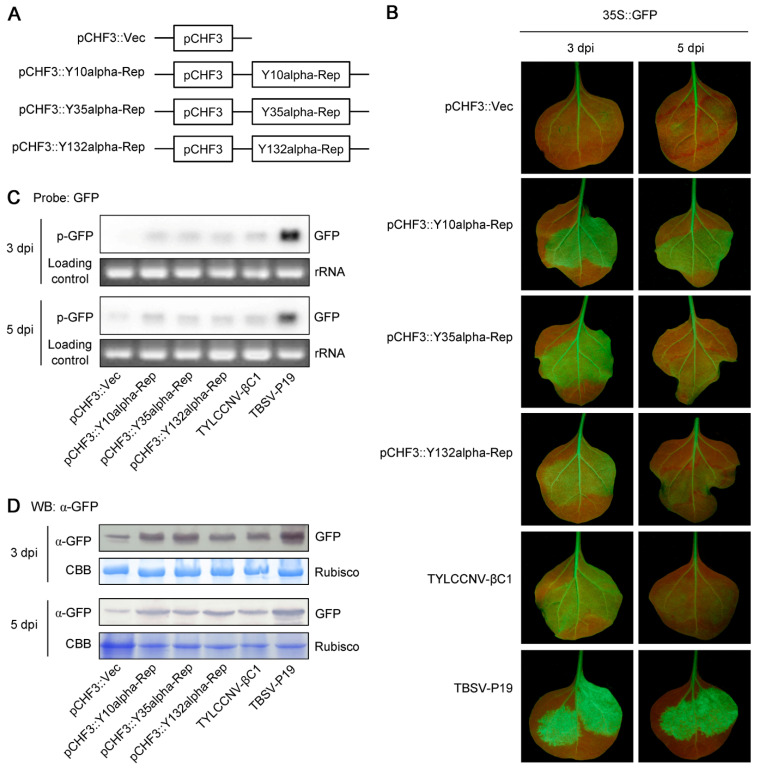
Alpha-Reps suppress the post-transcriptional gene silencing (PTGS) of local GFP. (**A**) Schematic diagram showing the constructs used in the local GFP PTGS assay in panel B. The ORFs of Y10alpha-Rep, Y35alpha-Rep, and Y132alpha-Rep were cloned into the binary vector, pCHF3, to be used for transient expression. An empty pCHF3 vector (pCHF3::Vec) was used as the negative control. (**B**) Suppression of PTGS of *GFP* in leaves of transgenic *N. benthamiana* 16c plants. Leaves of 16c plants were coagroinfiltrated with constructs harboring GFP (35S::GFP) and either a pCHF3::Vec, pCHF3::Y10alpha-Rep, pCHF3::Y35alpha-Rep, or pCHF3::Y132alpha-Rep. Leaves of 16c plants coagroinfiltrated with 35S::GFP and TYLCCNV-βC1 or TBSV-P19 were used as the positive controls. The agroinfiltrated leaves were photographed under UV light at 3 and 5 days post infiltration (dpi). (**C**) Northern blot analysis of GFP mRNA accumulation in agroinfiltrated leaf patches shown in panel B. An ethidium bromide-stained gel shown below the blots provides an RNA-loading control. (**D**) Western blot (WB) assay of GFP accumulation in agroinfiltrated leaf patches shown in panel B. CBB staining of the large subunit of Rubisco was used as a loading control. For (**B**–**D**), the experiments were repeated three times with similar results.

To determine the biological functions of these alpha-Reps, we first examine their ability to suppress GFP silencing in a GFP-expressing transgenic *N. benthamiana* line 16c. To this end, the ORFs of these three types of alpha-Reps were amplified by PCR and cloned into a pCHF3 vector to generate pCHF3::Y10alpha-Rep, pCHF3::Y35alpha-Rep, and pCHF3::Y132alpha-Rep constructs (Figure 2A). In the PTGS assay, *A. tumefaciens* containing a binary vector designed to transiently express sense GFP (35S::GFP) and *A. tumefaciens* harboring candidate suppressor genes were coinfiltrated into leaves of 16c plants as described in previous research [25]. In this study, 16c plants infiltrated with *A. tumefaciens* containing an empty pCHF3 vector (pCHF3::Vec) were used as negative controls and plants infiltrated with *A. tumefaciens* containing TBSV-P19 [44] or TYLCCNV-βC1 [43] were used as positive controls. As expected, leaves of 16c plants coinfiltrated with *A. tumefaciens* containing 35S::GFP, and TBSV-P19 or TYLCCNV-βC1 elicited relatively strong green GFP fluorescence due to suppression of GFP RNA silencing after incubation for 3–5 days (Figure 2B), especially those coinfiltrated with *A. tumefaciens* containing 35S::GFP and TBSV-P19. Similar to these positive controls, leaves coinfiltrated with *A. tumefaciens* containing 35S::GFP and pCHF3::Y10alpha-Rep, pCHF3::Y35alpha-Rep, or pCHF3::Y132alpha-Rep gave a strong green GFP fluorescence under UV light (Figure 2B). Compared with TBSV-P19, the Y10alpha-Rep, Y35alpha-Rep, and Y132alpha-Rep proteins showed weaker and different levels of suppressor activity on PTGS, which were stronger than that of TYLCCNV-βC1 (Figure 2B). These results indicated that all of the three types of alpha-Reps exhibited an RNA silencing suppressor activity.

To confirm the results observed under UV light, we determined the mRNA and protein levels of GFP using Northern blot and Western blot, respectively. As shown in Figure 2C, leaves infiltrated with *A. tumefaciens* containing 35S::GFP alone stopped fluorescing of GFP, which displayed the lowest mRNA level of GFP. In contrast, the mRNA level of GFP in leaves coinfiltrated with *A. tumefaciens* containing 35S::GFP and pCHF3::Y10alpha-Rep, pCHF3::Y35alpha-Rep, pCHF3::Y132alpha-Rep, and TYLCCNV-βC1 was higher than that in leaves infiltrated with *A. tumefaciens* containing 35S::GFP alone, but less than that in plants infiltrated with TBSV-P19. This result was consistent with the observations of fluorescence under UV light. Furthermore, the Western blot analysis displayed a similar result to the Northern blot analysis (Figure 2D). Taken together, Northern blot and Western blot analyses verified that the higher fluorescence observed in leaves of 16c plants coinfiltrated with the pCHF3::Y10alpha-Rep, pCHF3::Y35alpha-Rep, or pCHF3::Y132alpha-Rep, together with 35S::GFP, was due to the increased accumulation of GFP mRNA and protein. Collectively, these results suggest that three types of alpha-Reps are suppressors of the local GFP PTGS, but are weaker than TBSV-P19.

### 3.3. Alpha-Reps Are Suppressors of Systemic GFP PTGS

**Figure 3:**
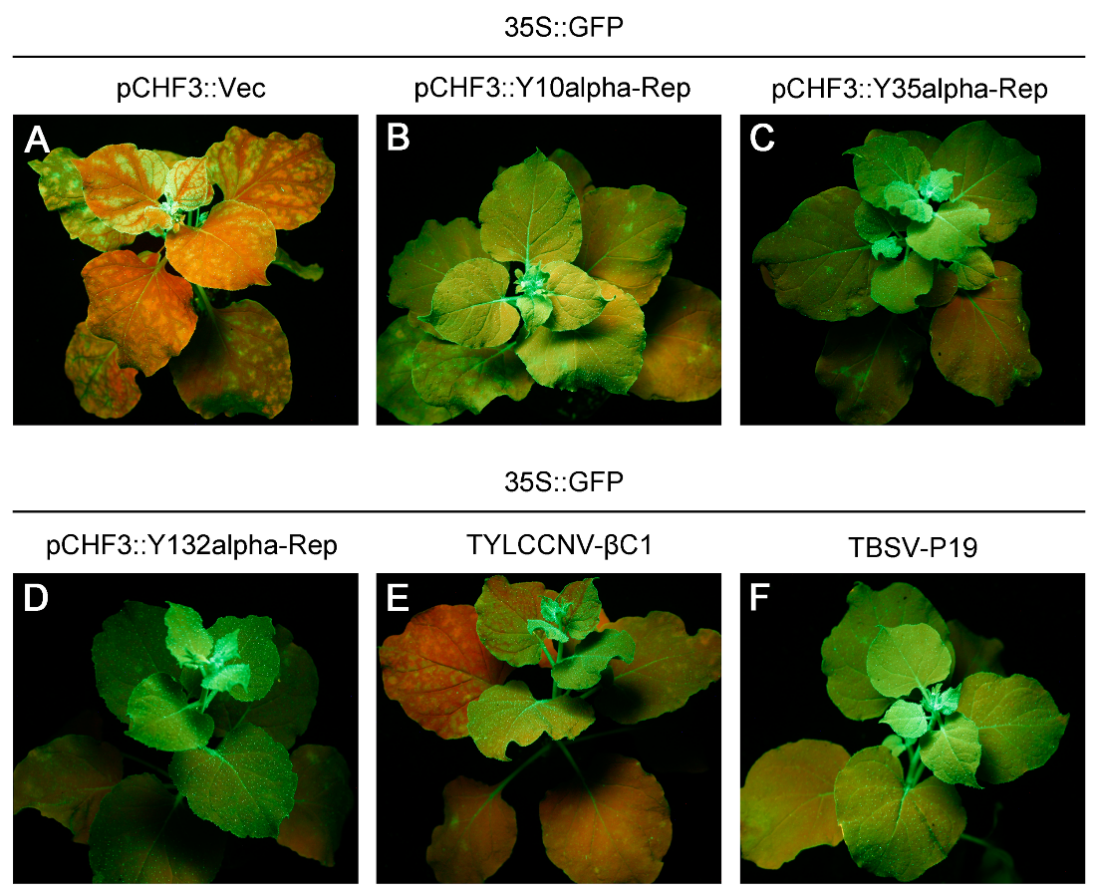
Alpha-Reps suppress the post-transcriptional gene silencing (PTGS) of systemic GFP. GFP fluorescence observed in transgenic *N. benthamiana* 16c plants agroinoculated with 35S::GFP plus pCHF3::Vec (**A**) pCHF3::Y10alpha-Rep (**B**) pCHF3::Y35alpha-Rep (**C**) pCHF3::Y132alpha-Rep (**D**) TYLCCNV-βC1 (**E**) or TBSV-P19 (**F**) at 30 days post infiltration (dpi). Leaves of 16c plants coagroinfiltrated with 35S::GFP plus pCHF3::Vec were used as the negative control and leaves of 16c plants coagroinfiltrated with 35S::GFP plus TYLCCNV-βC1 or TBSV-P19 were used as the positive controls.

Previous studies have reported that PTGS in plants is divided into local and systemic forms [45,46,47]. We next investigated the ability of these alpha-Reps to prevent the establishment of silencing induced by 35S::GFP in 16c *N. benthamiana* plants. Leaves of 16c plants were infiltrated with *A. tumefaciens* containing either 35S::GFP or 35S::GFP in combination with respective test constructs. The infiltrated plants were then monitored under a hand-held UV lamp at 10 dpi, 20 dpi, 30 dpi, and 40 dpi, respectively. At 10 dpi, 16c plants infiltrated with 35S::GFP plus pCHF3::Vec or TYLCCNV-βC1 showed the characteristic vein proximal GFP silencing in the new systemic leaves, and became almost entirely red at 30 dpi (Figure 3A,E). The statistical result showed that systemic GFP silencing occurred in 40–50% of the plants infiltrated with 35S::GFP plus pCHF3::Vec or TYLCCNV-βC1 at 10 dpi, and then increased to approximately 80% at 30 dpi (Table 1). In contrast, more than 70% of the 16c plants infiltrated with 35S::GFP plus pCHF3::Y35alpha-Rep or pCHF3::Y132alpha-Rep showed green fluorescence under UV illumination at 30 dpi, and even 40 dpi (Table 1 and Figure 3C,D), indicating that a suppression of GFP silencing occurred in the systemic leaves. More interestingly, all 16c plants infiltrated with 35S::GFP plus pCHF3::Y10alpha-Rep showed green fluorescence at 30 dpi under UV illumination, which was similar to plants infiltrated with 35S::GFP plus TBSV-P19 (Figure 3B,F), suggesting that the Y10alpha-Rep could effectively prevent the spread of GFP gene silencing in the infiltrated 16c plants. Taken together, these results suggest that these three types of alpha-Reps are suppressors of PTGS and capable of restraining systemic GFP gene silencing in 16c *N. benthamiana* plants.

### 3.4. Alpha-Reps Can Reverse Established Methylation-Mediated TGS

**Figure 4:**
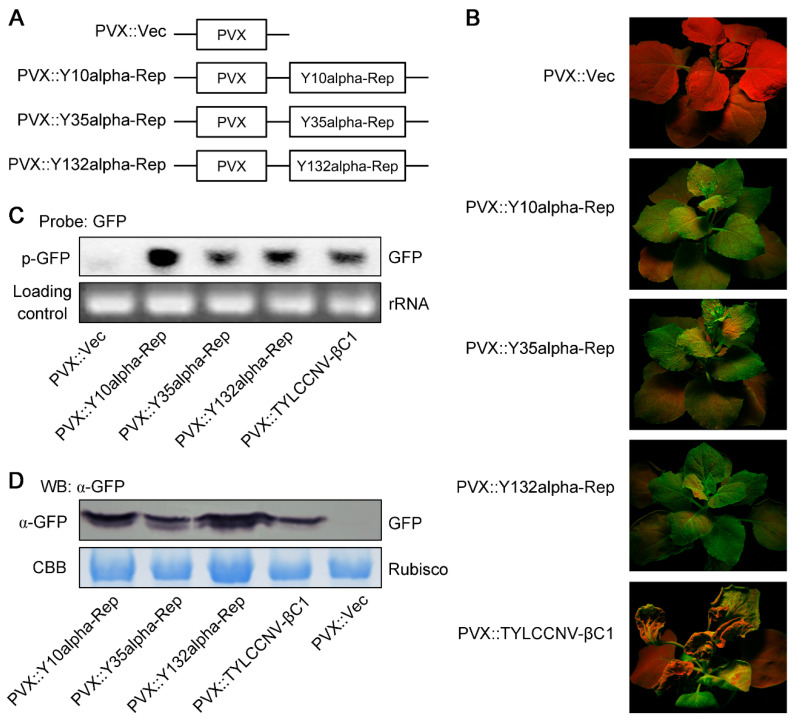
Alpha-Reps suppress the transcriptional gene silencing (TGS) of GFP. (**A**) Schematic diagram showing the constructs used in the GFP TGS assay in panel B. The ORFs of Y10alpha-Rep, Y35alpha-Rep, and Y132alpha-Rep were cloned into the PVX, to be used for transient expression. An empty PVX vector (PVX::Vec) was used as the negative control. (**B**) Transgenic *N. benthamiana* 16-TGS plants were agroinfiltrated with PVX::Y10alpha-Rep, PVX::Y35alpha-Rep, or PVX::Y132alpha-Rep, and the plants were photographed under UV light at 14 dpi. 16-TGS plants agroinoculated with PVX::Vec and PVX::TYLCCNV-βC1 were used as negative and positive controls, respectively. (**C**) Northern blot analysis of GFP mRNA accumulation in agroinfiltrated leaf patches is shown in panel B. An ethidium bromide-stained gel shown below the blots provides an RNA-loading control. (**D**) Western blot (WB) assay of GFP accumulation in agroinfiltrated leaf patches shown in panel B. CBB staining of the large subunit of Rubisco was used as a loading control. For (**B**–**D**) the experiments were repeated three times with similar results.

More recently, a study showed that the alpha-Rep encoded by Cotton leaf curl Multan alphasatellite (CLCuMuA) can restore the expression of a transcriptionally silenced GFP transgene in *N.* *benthamiana* [48]. Keeping this point in mind, we next investigated whether alpha-Reps identified from Yunnan province of China have the ability to suppress methylation-mediated TGS using 16-TGS plants, which contain a transcriptionally silenced GFP transgene flanked by the 35S promoter [25,36]. For this propose, the ORFs of Y10alpha-Rep, Y35alpha-Rep, and Y132alpha-Rep were cloned into a Potato virus X (PVX)-based vector [42] (Figure 4A) and then infiltrated into the 16-TGS plants as described in previous research [25]. As shown in Figure 4B, 16-TGS plants infiltrated with PVX::Y10alpha-Rep, PVX::Y35alpha-Rep, or PVX::Y132alpha-Rep displayed strong green GFP fluorescence as a consequence of the suppression of GFP RNA silencing, while plants infiltrated with PVX::Vec alone showed only red fluorescence under UV illumination at 14 dpi. This result was further confirmed by Northern blot and Western blot analyses. As expected, Northern blot and Western blot analyses showed that the presence of GFP fluorescence in 16-TGS plants infected with PVX::Y10alpha-Rep, PVX::Y35alpha-Rep, or PVX::Y132alpha-Rep was due to the accumulation of GFP mRNA and protein (Figure 4C,D). These results suggest that all the three types of alpha-Reps identified from Yunnan province, China can efficiently reverse established methylation-mediated TGS in *N. benthamiana* plants.

### 3.5. Alpha-Reps Can Enhance the Accumulation of Their Helper Virus

**Figure 5:**
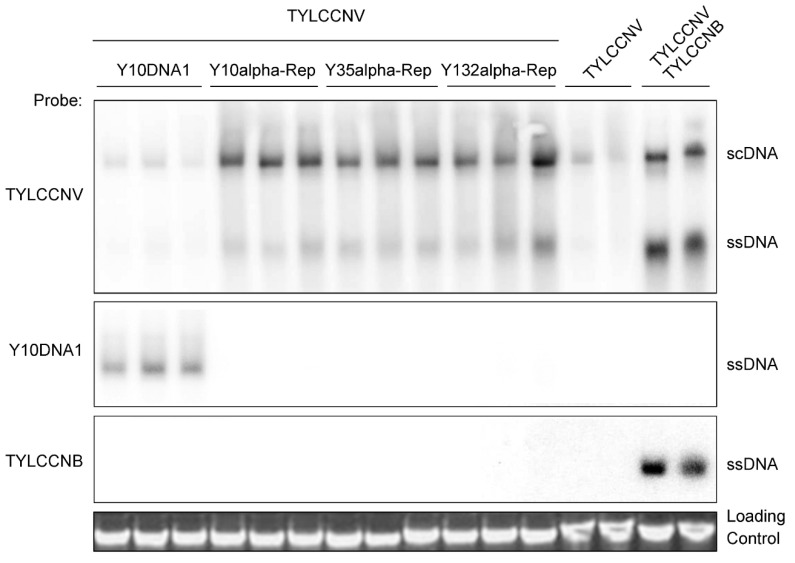
Southern blot analysis of viral DNA accumulation in *N. benthamiana* plants infected by TYLCCNV alone or together with Y10DNA1, Y10alpha-Rep, Y35alpha-Rep, Y132alpha-Rep, or TYLCCNB at 30 dpi. Total genomic DNA (approximately 10 µg for each lane) from a mixture of three seedlings was used for the Southern blot. Blots were probed with the coat protein gene sequence of TYLCCNV (top), the Rep of Y10DNA1 (middle), and the full-length sequence of TYLCCNB (bottom). An ethidium bromide-stained gel shown below the blots provides a DNA-loading control (downmost). The positions of supercoiled (scDNA) and single stranded (ssDNA) forms are indicated, respectively.

Our and Fauquet’s groups have previously showed that the whole alphasatellites can suppress the accumulation of their helper viruses during coinfection with begomovirus/betasatellite complexes [30,33]. We next asked whether, as suppressors, the alpha-Reps can interfere with the accumulation of their helper viruses during coinfection. To examine this, we determined the viral DNA accumulation in *N. benthamiana* plants infected by TYLCCNV alone or together with Y10 alphasatellite (Y10DNA1), Y10alpha-Rep, Y35alpha-Rep, Y132alpha-Rep, or TYLCCNB at 30 dpi. Using TYLCCNV as probe, the TYLCCNV DNA accumulation appeared in *N. benthamiana* plants infected by TYLCCNV alone, and the DNA accumulation was reduced when co-infected by TYLCCNV and Y10DNA1, as in a previous study [33]. While coinfection of TYLCCNV with the alpha-Reps showed increased DNA accumulation when compared with *N. benthamiana* plants infected by TYLCCNV alone, the highest DNA accumulation was tested in co-infected by TYLCCNV and TYLCCNB. The Y10DNA1 accumulation was only detected in plants co-infected by TYLCCNV and Y10DNA1, and the TYLCCNB accumulation only showed in coinfection of TYLCCNV and TYLCCNB (Figure 5). This result indicates that alpha-Reps had a potential capacity to enhance the accumulation of their helper virus, and that they may have different models of action compared with the whole alphasatellites during viral infection.

## 4. Discussion

The pathogenicity of helper viruses varies widely among different begomovirus/betasatellite/alphasatellite complexes. In the TYLCCNV/TYLCCNB disease complex associated with Y10 alphasatellite, the helper virus TYLCCNV must rely on TYLCCNB to produce typical symptoms such as leaf blade rolling, vein thickening, yellow veins, stem distortion, plant dwarfing, and enation [21,49]. However, in the Y35 alphasatellite-accompanied TbCSV/TbCSB and the Y132 alphasatellite-accompanied TbCSV/AYVB disease complexes, the helper virus TbCSV alone can infect *N. benthamiana* and cause severe upward curling, leaf vein thickening, shoot curling, and plant dwarfing. In the presence of betasatellites, these disease complexes would induce more severe symptoms during viral infections [50]. In the Cotton leaf curl Rajasthan virus (CLCuRaV)/Cotton leaf curl Multan betasatellite (CLCuMuB), the disease complex associated with GDarSLA and GMusSLA, the helper virus CLCuRaV alone is also not able to cause typical symptoms or systemic infection, while severe symptoms such as leaf downward curling, narrower new leaves, and stalk distortion were only induced by co-infected with CLCuRaV and CLCuMuB [30,34].

RNA silencing is an important mechanism for plants to defend against viruses, while plant viruses can encode various PTGS suppressors to withstand host gene silencing and ultimately achieve successful systemic infections of their hosts. It has been shown that the alpha-Reps encoded by GDarSLA and GMusSLA have a similar silencing-suppressor activity as TBSV-P19, which is stronger than the βC1 encoded by the betasatellites and the C2, C4 or V2 encoded by the helper viruses [30]. In this study, we found that the silencing-suppressor activity of Y10alpha-Rep is stronger than that of the TYLCCNB-βC1, which is characterized in our previous study [24]; that the silencing-suppressor activity of Y35alpha-Rep is stronger than that of the TYLCCNB-βC1 in the early infection (3 dpi) and then followed by TYLCCNB-βC1; and that the silencing-suppressor activity of Y132alpha-Rep is similar to that of the TYLCCNB-βC1 during infection. Sequence analysis revealed that each of these three types of alphasatellites encode a 315 amino acid alpha-Rep protein with a homology of 90.0–93.0% (Table S2). They showed a homology of 74.0–77.0% and 52.0–53.0% to alpha-Reps encoded by GDarSLA and GMusSLA, respectively (Table S2). This indicates that the activity of the suppressors is not related to the protein sequence. Our experiments also found that all three alpha-Reps can suppress systemic silencing as well as local silencing (Figure 3), and that Y10alpha-Rep has a similar strong ability to inhibit systemic silencing to the positive control TBSV-P19, and inhibits systemic silencing even after 40 days of infiltration.

In the begomovirus/betasatellite/alphasatellite disease complex, each of these can encode silencing suppressors; however, the silencing pathways and mechanisms that are employed by these suppressors are not identical. Some viruses encode multiple RNA silencing suppressors. Amin et al. reported that the CLCuMuV/CLCuMuB complex is capable of encoding V2, C2, C4, and βC1 proteins with gene silencing suppressor activity, and they may work synergistically [51]. Coincidentally, Vanitharani et al. found that when simultaneously infected with the Cameroon strain of African cassava mosaic virus (ACMV-[CM]) and East African cassava mosaic Cameroon virus (EACMCV), plants would display synergistic severe mosaic disease [52]. This is because ACMV-[CM] AC4 and EACMCV AC2 are able to work synergistically, suppressing the PTGS of their hosts and causing a dramatic increase in the symptom severity and viral DNA accumulation of both viruses. Here, we suspect that in the begomovirus/betasatellite/alphasatellite disease complex, the two suppressors (alpha-Rep and βC1) encoded by betasatellites and alphasatellites may act at distinct steps in the RNA silencing pathway and synergistic interaction, and ultimately help the viral complex to systemically infect the host plant to achieve a coexisting and balanced environment in the disease complex-host plant.

Since the genome of geminiviruses undergoes rolling circle replication in the nucleus through double-stranded DNA intermediates that associate with cellular histone proteins to form minichromosomes, plants can use the TGS pathway to protect against geminivirus infection [53,54,55,56]. It has been shown that L2 encoded by Beet curly top virus (BCTV) [57], C2 encoded by Beet severe curly top virus (BSCTV) [58], Rep encoded by Tomato yellow leaf curl Sadinia virus (TYLCSV) [59], V2 encoded by Tomato yellow leaf curl virus (TYLCV) [60], CLCuMuV-C4 [11], C4 encoded by Tomato leaf curl New Delhi virus (ToLCNDV) [61], Tomato leaf curl Yunnan virus (TLCYnV) [62], and TYLCCNB-βC1 can suppress the methylation pathway and TGS [25,37]. In the present study, we found that three types of alpha-Reps have a similar function to TYLCCNB-βC1, which not only has PTGS suppressor activity, but also has TGS suppressor activity (Figure 2 and Figure 4). Our results also suggest that the Y10alpha-Rep has the strongest PTGS and TGS suppressor activity among the three alpha-Reps identified in Yunnan, China (Figure 2, Figure 3 and Figure 4), and that Y10 is the main type of alphasatellite found in China. To the best of our knowledge, this is the first report in which alpha-Reps identified in China have been found to have TGS suppressor activity. However, the suppressor mechanisms of PTGS and TGS need to be further studied.

To date, most of the RNA silencing suppressors are found to belong to viral pathogenicity factors, replication enhancers, and move proteins, which are not required for viral replication but can affect viral movement or accumulation [46,63]. Alphasatellites are always associated with the begomovirus/betasatellite complex in China, and the contribution of alphasatellites to geminivirus infection remains unknown. Our previous study showed that alphasatellites may act to attenuate the symptoms induced by the begomovirus/betasatellite complex through reducing the accumulation of the helper virus and betasatellites. Our results showed that three types of alpha-Reps could all significantly increase the accumulation of their helper virus (Figure 5). We speculated that when co-inoculated alpha-Reps with their helper virus TYLCCNV, alpha-Reps could suppress host RNA silencing due to their function as PTGS and TGS suppressors, and thereby help their helper virus to infect the host plants and increase the helper virus accumulation. However, when hosts co-inoculated an intact alphasatellite (Y10DNA1) with its helper virus TYLCCNV, the accumulation of the helper virus was reduced (Figure 5). This may be because under the co-existence of alphasatellite and its helper virus condition, alpha-Rep first needs to function as a replicating protein to achieve the replication of alphasatellites and then acts as a PTGS and TGS suppressor. In this case, the helper virus has to compete with the alphasatellite to utilize the host plant’s replication system and cellular environments for its replication. Thus, in the presence of alphasatellites, the accumulation of the helper virus was decreased due to the sharing of host cell resources with alphasatellites. This is consistent with our previous report showing that alphasatellites are capable of reducing the accumulation of helper viral genomic DNA during begomovirus/betasatellite infection [33]. Our results regarding the biological functions of alpha-Reps may provide a new idea for understanding alphasatellites and their disease complexes.

## 5. Conclusions

In summary, our results indicate that alpha-Reps from alphasatellites in Yunnan province in China have substantial suppressor activities on host PTGS and TGS, which may overcome host defense and increase the accumulation of their helper virus during coinfection.

## Figures and Tables

**Table 1:** Suppression of systemic GFP silencing by alpha-Reps in transgenic *N. benthamiana* 16c plants at different days post infiltration (dpi).

Treatments	10 dpi ^a^	20 dpi ^a^	30 dpi ^a^	40 dpi ^a^
35S::GFP + pCHF3::Vec	9/24	15/24	19/24	19/24
35S::GFP + pCHF3::Y10alpha-Rep	0/36	0/36	0/36	0/36
35S::GFP + pCHF3::Y35alpha-Rep	4/36	4/36	5/36	5/36
35S::GFP + pCHF3::Y132alpha-Rep	1/24	3/24	7/24	7/24
35S::GFP + TYLCCNV-βC1	10/20	13/20	16/20	18/20
35S::GFP + TBSV-P19	0/24	0/24	0/24	0/24

^a^ Data are the number of plants showing systemic GFP silencing/total number of infiltrated plants.

## Data Availability

Not applicable.

## Supplementary Materials

The following are available online at https://www.mdpi.com/article/10.3390/v14020222/s1, Table S1. List of primers used in this study. Table S2. The homology of amino acid sequence among the alpha-Reps encoded by different alphasatellites.
